# Fluorescein sodium as a marker for focused ultrasound-induced blood-brain barrier disruption: a case report in a porcine model

**DOI:** 10.3389/fsurg.2025.1559195

**Published:** 2025-04-01

**Authors:** Yuan Xu, Thomas J. On, Mark C. Preul

**Affiliations:** The Loyal and Edith Davis Neurosurgical Research Laboratory, Barrow Neurological Institute, St. Joseph’s Hospital and Medical Center, Phoenix, AZ, United States

**Keywords:** fluorescence imaging, fluorescein sodium, focused ultrasound, blood-brain barrier disruption, confocal laser endomicroscopy (CLE), liquid biopsy, targeted drug delivery

## Abstract

Transcranial low-intensity focused ultrasound (FUS) enables noninvasive, targeted, and reversible blood-brain barrier (BBB) disruption, facilitating drug delivery and liquid biopsy of the brain. Using fluorescein sodium (FNa) with macroscopic widefield fluorescence and microscopic confocal laser endomicroscopy (CLE) imaging, we assessed BBB permeability after applying a frameless, electromagnetic-guided FUS system in a porcine model and confirmed with established MRI protocol and conventional histology. Both macroscopic and microscopic FNa fluorescence imaging findings correlated with contrast-enhanced MRI, providing direct evidence of BBB disruption. This approach demonstrates the utility of FNa for evaluating BBB permeability in preclinical studies.

## Introduction

Assessing blood-brain barrier (BBB) disruption necessitates reliable methods to confirm targeted permeability changes and evaluate tissue effects. Contrast-enhanced MRI, a common approach, utilizes gadolinium's ability to cross the disrupted BBB and enhance imaging of affected regions (1). However, while conventionally used, this approach depends on indirect digital reconstruction of MRI signals and lacks image resolution at the cellular level. Alternatively, fluorescein sodium (FNa) is a safe, low-molecular-weight fluorophore administered intravenously that typically does not penetrate an intact BBB but has been demonstrated to cross a disrupted BBB under various pathological conditions (2, 3). This property makes FNa an excellent option for directly visualizing BBB disruption in areas where the barrier has been compromised. In the surgical setting, its fluorescence can be detected with a widefield operating microscope and confocal laser endomicroscopy (CLE), a new FDA-approved cellular-level imaging system (4–6).

Transcranial low-intensity focused ultrasound (FUS), paired with intravenously injected microbubbles, allows for noninvasive, reversible, and targeted disruption of the blood-brain barrier (BBB) with millimeter precision (7). This technique utilizes FUS-induced cavitation, where microbubbles oscillate and produce mechanical forces on the vasculature, temporarily increasing BBB permeability (8). This effect typically lasts from hours to a few days, enabling the controlled delivery of therapeutic agents to specific brain regions. Preclinical and clinical studies have demonstrated the safety and therapeutic potential of FUS-mediated BBB modulation for improving drug delivery to the brain and enabling liquid biopsy of the brain in various neurological and neurodegenerative diseases (9–14).

This study describes an experimental case involving a prototype portable FUS system designed to induce BBB disruption (15) in a porcine model in which intravenous FNa was used to assess changes in BBB permeability in the area of FUS-induced disruption. The brain was evaluated macroscopically with widefield operating microscope imaging and microscopically with CLE, both detecting the FNa-induced fluorescence. The fluorescence imaging results were correlated with gadolinium-enhanced MRI and histological examination to characterize the extent of BBB disruption more comprehensively.

## Methods

### Microbubble formulation and delivery

Lumason (Bracco, Princeton, NJ, USA), a sulfur hexafluoride lipid-type microsphere suspension, served as the microbubble contrast agent. It was activated according to the manufacturer's instructions by adding 5 ml of saline to the vial, shaking vigorously for 20 s, and then withdrawing the required amount into a syringe. The entire vial of suspension was then diluted with 55 ml of sterile saline, resulting in a total of 60 ml of microbubble solution. This solution was infused intravenously at a rate of 4 ml/min. The infusion started 2 min before FUS initiation and continued until a total volume of 8 ml was delivered. FUS was initiated immediately after reaching this volume to ensure optimal microbubble circulation during sonication.

### Focused ultrasound procedure

One Yorkshire pig (Premier BioSource, Ramona, CA, USA) was utilized for the focused ultrasound procedure. The study involving animal participants were reviewed and approved by St. Joseph's Hospital and Medical Center Institutional Animal Care and Use Committee. General anesthesia was induced with an intramuscular injection of 0.6 ml/10 kg Telazol (50 mg tiletamine and 50 mg zolazepam per ml) and maintained with inhaled isofluorane at 2.5% and 3.5–4.5 L/min flow rate through an endotracheal tube. Two-millimeter head CT scans and one-millimeter contrast-enhanced T1-weighted and T2-weighted MRI scans were conducted one week prior to the FUS procedure for skull and brain 3D reconstruction and trajectory planning. The FUS system (Cordance Medical, Mountain View, CA, USA) employs a frameless neuronavigation approach with a permanent magnet-based tracking. A signal generator generates an electromagnetic field for real-time sensor tracking, with sensors positioned on the ultrasound transducer and the subject.

CT and MRI datasets were fused to create a 3D model, highlighting the region of interest (ROI) and facial landmarks. The FUS system then identified the optimal transducer position based on the shortest and most effective route to the ROI. Registration between the tracking system's coordinate space and the CT/MRI coordinates was established to align the subject's anatomy with the image data. Finally, the transducer, equipped with tracking sensors, was positioned according to these coordinates. An error metric guided the operator to minimize deviations from the planned location, ensuring precise alignment with the ROI.

The pig received gadolinium contrast immediately before the FUS procedure. Following sonification, contrast-enhanced T1W and T2W MRI scans were performed and compared with the pre-procedural T1W and T2W MRI scans. The ultrasound settings were 250 kHz frequency, 10 ms pulse duration, 1 Hz pulse repetition frequency, 1% duty cycle, and *in situ* focal pressures of 0.275–0.325 MPa.

### Brain specimen harvest and processing

The first dose of FNa was administered intravenously at a dosage of 5 mg/kg immediately following the FUS procedure. After MRI imaging, a bilateral craniectomy was performed to expose the entire brain. The dura was opened bilaterally with a C-shaped incision and reflected toward the midline to reveal both hemispheres. A second dose of 5 mg/kg of FNa was given intravenously after the brain was exposed. Then, the swine was euthanized with a 10 ml intravenous bolus of Euthazol (390 mg pentobarbital sodium and 50 mg phenytoin sodium per ml). Following the euthanization of the swine, both cerebral hemispheres were separated from the brain stem and harvested. They were placed in a 3D-printed brain matrix and sectioned coronally into slices that were 0.5 cm thick. The slices were laid flat on a tray covered with a moist drape for fluorescence imaging.

### Widefield fluorescence imaging

After the brain was exposed, it was imaged with a Zeiss Kinevo 900 surgical microscope (Carl Zeiss Meditec AG, Oberkochen, Germany) using the YELLOW 560 fluorescence module. The lowest magnification and working distance settings were selected to maximize fluorescence signal detection. After the brain was harvested and sectioned, each slice affected by the FUS trajectory was imaged using YELLOW 560 under the same magnification and working distance settings.

### Confocal laser endomicroscopy imaging

Confocal laser endomicroscopy (CLE) imaging was performed using a ViewnVivo CLE imaging system (Optiscan Imaging Ltd., Melbourne, Australia). The imaging probe was positioned perpendicular to the tissue surface in a holder and maintained in firm contact with the tissue. Areas displaying positive widefield fluorescence were scanned with the CLE probe. Optimal imaging parameters were chosen based on the established parameters from the previous studies using an older generation of the same CLE imaging platform (16). The highest imaging resolution of 1,920 × 1,080 was selected for the 464 × 261 µm field of view, resulting in 1.3 s per image scanning speed. Band pass 515–575 filter was chosen for best image contrast. Laser power was adjusted to 70%–100% of the maximum output (1,000 µW), and the gain was set to 2,400 to enhance image quality. Normal brain tissue that was unaffected by FUS and negative for widefield fluorescence was also scanned using CLE.

### Histology analysis

After widefield fluorescence and CLE imaging, the areas with positive FNa fluorescence were sampled with the surrounding normal brain tissue. Normal brain tissue was also sampled as a control for histology processing. Sampled tissues were preserved in 10% neutral buffered formalin. After routine processing and paraffin embedding, all brain tissue samples were cut into 3 μm sections and stained with hematoxylin and eosin.

## Results

**Figure 1:**
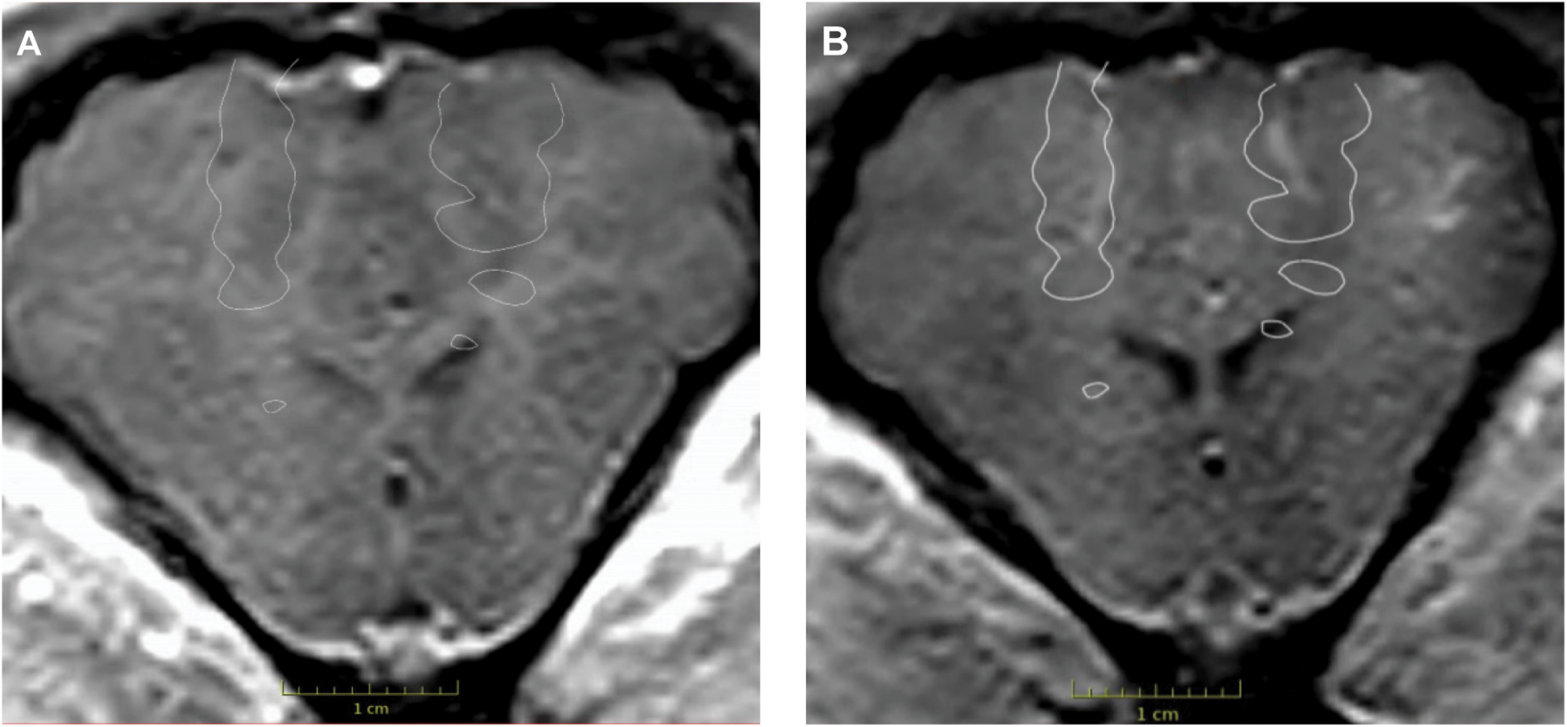
Contrast-enhanced T1W MRI images were acquired before and after the FUS procedure. Contrast enhancement was absent on the pre-procedural MRI **(A)** but present bilaterally on the post-procedural MRI **(B)** The white outlines in both images indicate the planned target for the FUS procedure. Used with permission from Barrow Neurological Institute, Phoenix, Arizona.

FUS sonication was successfully conducted in the frontal lobes near the motor cortex on both sides. Immediately after the FUS procedure, MRI imaging of the pig was performed. Post-sonication T1W MRI sequences showed contrast enhancement in the areas targeted by the FUS beams, which was not seen in the pre-sonication MRI images (Figure 1).

**Figure 2:**
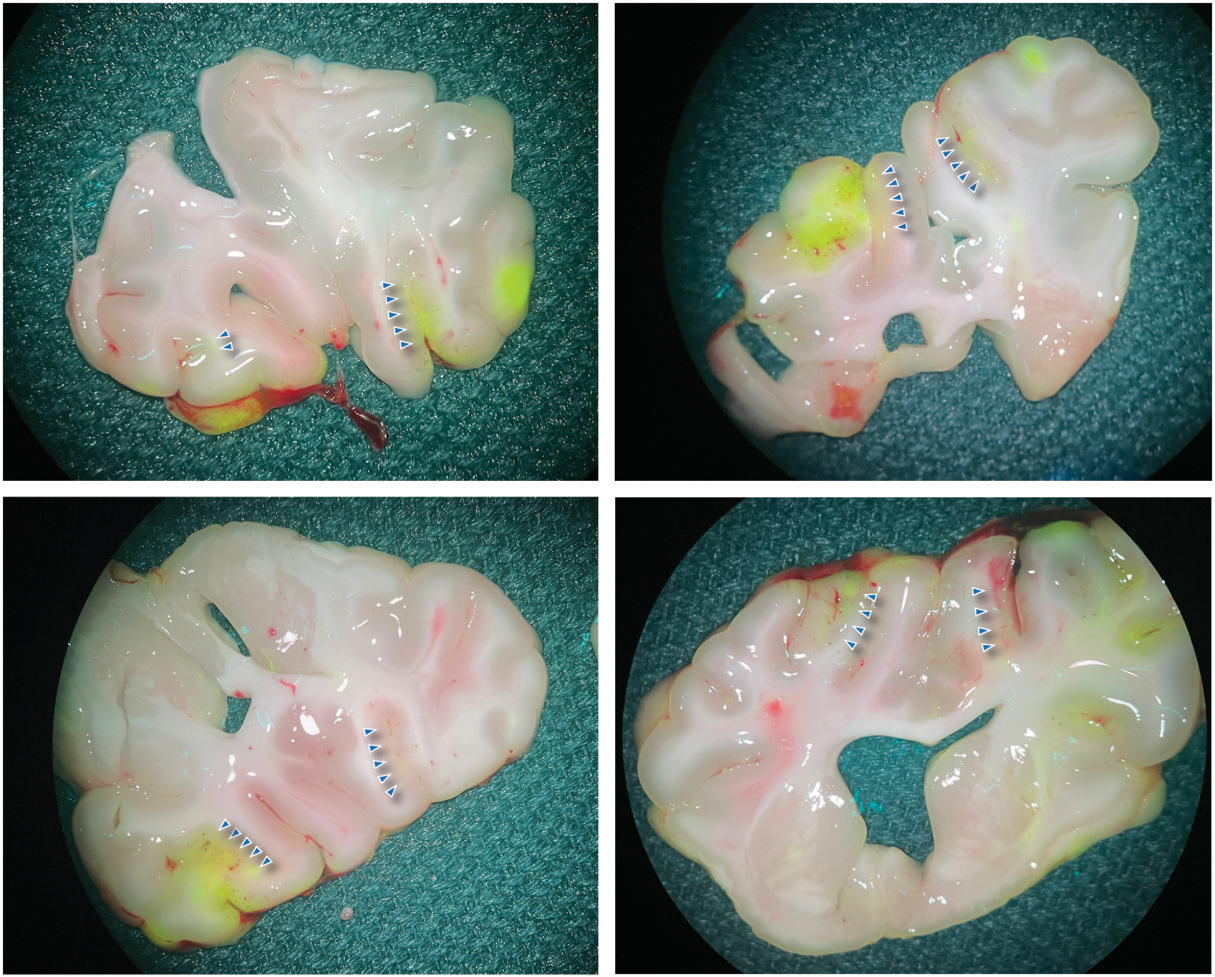
Widefield FNa fluorescence images of the brain. The brain was sectioned coronally to expose the sonicated areas. The blue arrowheads indicate the area with positive fluorescence. Used with permission from Barrow Neurological Institute, Phoenix, Arizona.

Afterward, the brain was exposed through a bilateral craniectomy, and a second dose of FNa was administered before the swine was euthanized. The brain was then harvested, coronally sliced, and examined using the widefield yellow fluorescence module on a surgical microscope. Fluorescence imaging was utilized to complement and confirm the MRI findings. The fluorescence signal observed with the widefield yellow fluorescence module enabled the localization of disrupted regions. Strong, specific fluorescence was visibly noted in areas corresponding to those targeted by FUS (Figure 2). CLE and histological analysis further validated these findings.

**Figure 3:**
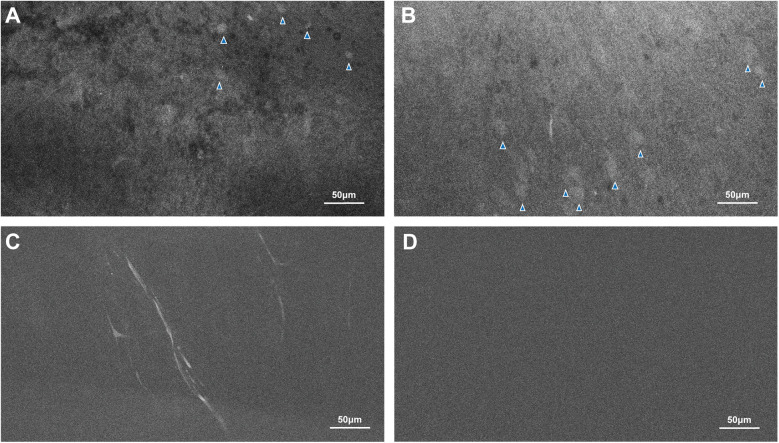
CLE images revealed evidence of the FUS effect. The fluorescence signals indicated by the blue arrowheads in **(A,B)**, as well as small caliber capillaries seen in **(C)**, are present in FUS-treated areas but not in areas not affected by FUS **(D)**. Used with permission from Barrow Neurological Institute, Phoenix, Arizona.

**Figure 4:**
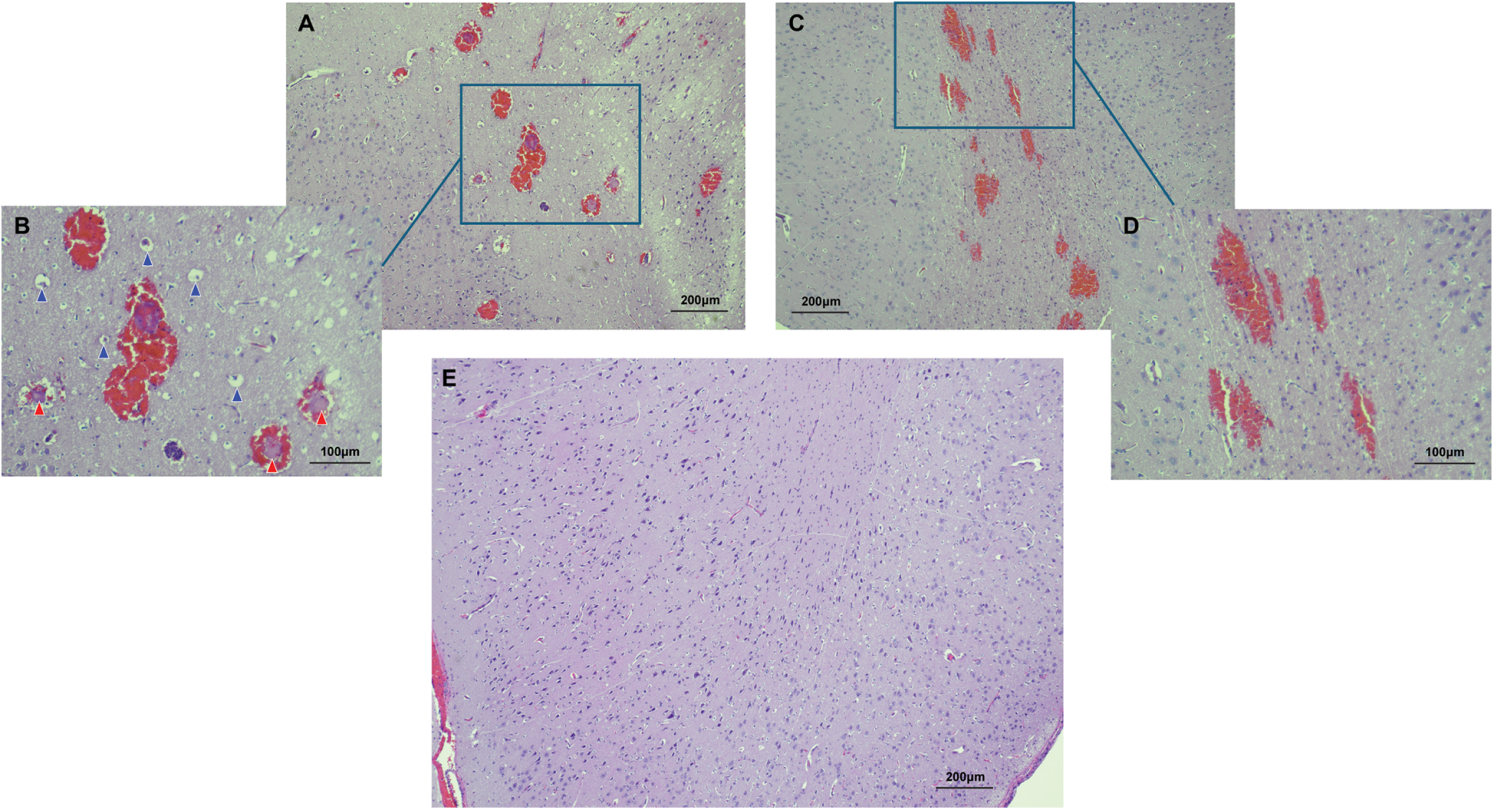
Histological features of the FUS effect from two areas are shown. In the first area **(A,B)**, perivascular extravasations of red blood cells were observed, indicating successful BBB disruption. High power magnification further revealed intravascular fibrin deposits (indicated by red arrowheads) and cellular vacuolization (indicated by blue arrowheads). In the second area **(C,D)**, perivascular extravasations were present, but no intravascular deposits nor cellular vacuolization was seen. No histological evidence of FUS effect was identified in normal non-sonicated areas of the brain **(E)**. Used with permission from Barrow Neurological Institute, Phoenix, Arizona.

Microscopic analysis using CLE demonstrated that regions of high fluorescence observed correlatively corresponded to cellular vacuolization and vascular involvement in the FUS-targeted areas. Signals were detected from vacuolated cells and blood vessels (Figures 3A–C). In contrast, CLE imaging of normal brain tissue exhibited no signal, consistent with the absence of FNa in non-sonicated brain tissue (Figure 3D). Histological examination validated these findings, revealing red blood cell extravasation in the sonicated regions with no indication of significant vascular or cellular injury (Figures 4A–D). Non-sonicated areas appeared normal, showing no signs of vascular or cellular changes (Figure 4E).

## Discussion

FNa, as an imaging marker, provides information about the spatial accuracy of FUS-induced changes. Under normal conditions, FNa does not cross intact BBB. Thus, FNa fluorescence in brain tissue detected by the widefield surgical microscope and CLE that would otherwise not be present directly indicates the BBB permeability changes at both gross and microscopic levels (3). This aids in refining sonication parameters and optimizing drug delivery and liquid biopsy strategies. Previous preclinical studies applied FNa-based CLE imaging to evaluate brain microcirculation and injury-induced BBB change (17). In our experimental setting, fluorescence imaging was conducted ex vivo after the euthanasia of the swine and extraction of the brain because we aimed to image the entire area affected by FUS that extended deep beyond the brain's surface. Although this scenario describes ex vivo FNa imaging, *in vivo* imaging of FUS targets may be feasible. Live imaging, particularly through widefield and especially by CLE imaging of deeper brain regions, could trace the path of FUS and enable analysis of tissue effects. This would require careful surgical hemostasis, as active bleeding may obscure FNa localization due to the presence of erythrocyte masses. Incorporating FNa into preclinical workflows for *in vivo* imaging could enhance our approaches to understanding BBB permeability and guide the development of more effective, targeted interventions.

FNa fluorescence imaging with both widefield and CLE has been performed *in situ* in real time during surgeries after the adequate exposure of the surgical target. Following intravenous administration, FNa rapidly circulates systemically, and its fluorescence signal can be detected within seconds, making it ideal for evaluating BBB disruption in a surgical setting without procedural delays. Widefield fluorescence imaging reveals FNa accumulation in exposed tissue, whereas CLE offers high-resolution microscopic visualization upon direct tissue contact. Both imaging modalities yield results in real time without the need for postprocessing.

Our effort was to showcase the effect of the FUS procedure on the BBB using fluorescence imaging in addition to the established MRI protocol. The microbleeding seen in the histology is consistent with previous reports of histological changes after FUS treatment and not associated with significant insult to the brain tissue (18, 19). The FUS-induced cellular effects were microscopic and confined to the sonication channel. Although patchy enhancement was noted in one FUS-targeted region on MRI, our widefield and confocal examinations did not reveal fluorescence patterns that correspond to the appearance of the MRI signal. Instead, FNa extravasation appeared consistent across both targeted sites, suggesting potential modality differences or MR imaging artifacts.

Being a technical report with observations derived from a single porcine model, this study has limitations. The study only showed acute BBB disruption without evaluating longer-term outcomes such as tissue recovery or sustained permeability changes. The potential impact of prolonged anesthesia on histological findings cannot be excluded. Physiological changes may influence tissue characteristics and the interpretation of results, although the animal remained within normal cardiorespiratory parameters.

## Conclusions

These findings demonstrate that FNa offers a direct and reliable means of visualizing FUS-induced BBB disruption at both gross and microscopic levels. This complements traditional MRI-based approaches by allowing more direct identification of permeability changes. The improved visualization can help refine therapeutic strategies that leverage FUS for targeted drug delivery or liquid biopsy.

## Data Availability

The raw data supporting the conclusions of this article will be made available by the authors, without undue reservation.

## References

[1] Rezai AR Ranjan M D'Haese PF Haut MW Carpenter J Najib U Noninvasive hippocampal blood-brain barrier opening in Alzheimer’s disease with focused ultrasound. Proc Natl Acad Sci U S A. (2020) 117(17):9180–2. 10.1073/pnas.2002571117 32284421 PMC7196825

[2] Ahishali B Kaya M. Evaluation of blood-brain barrier integrity using vascular permeability markers: Evans blue, sodium fluorescein, albumin-Alexa fluor conjugates, and horseradish peroxidase. Methods Mol Biol. (2021) 2367:87–103. 10.1007/7651_2020_316 32785841

[3] On TJ Xu Y Abramov I Alcantar-Garibay O Preul MC. Letter to the editor. Confocal laser endomicroscopy of fluorescein uptake in the brain tumor microenvironment. J Neurosurg. (2024) 141(6):1752–4. 10.3171/2024.5.JNS24967 39241260

[4] Xu Y On TJ Abramov I Restelli F Belykh E Mathis AM Intraoperative *in vivo* confocal endomicroscopy of the glioma margin: performance assessment of image interpretation by neurosurgeon users. Front Oncol. (2024) 14:1389608. 10.3389/fonc.2024.1389608 38841162 PMC11151089

[5] Abramov I Mathis AM Xu Y On TJ Belykh E Mignucci-Jimenez G Intraoperative confocal laser endomicroscopy during 5-aminolevulinic acid–guided glioma surgery: significant considerations for resection at the tumor margin. J Neurosurg. (2025) 142(2):429–42. 10.3171/2024.5.JNS24140 39332037

[6] Höhne J Schebesch K-M de Laurentis C Akçakaya MO Pedersen CB Brawanski A Fluorescein sodium in the surgical treatment of recurrent glioblastoma multiforme. World Neurosurg. (2019) 125:e158–64. 10.1016/j.wneu.2019.01.024 30682505

[7] Meng Y Kalia LV Kalia SK Hamani C Huang Y Hynynen K Current progress in magnetic resonance-guided focused ultrasound to facilitate drug delivery across the blood-brain barrier. Pharmaceutics. (2024) 16(6):719. 10.3390/pharmaceutics16060719 38931843 PMC11206305

[8] Chen H Anastasiadis P Woodworth GF. MR imaging-guided focused ultrasound-clinical applications in managing malignant gliomas. Magn Reson Imaging Clin N Am. (2024) 32(4):673–9. 10.1016/j.mric.2024.05.006 39322356 PMC13067882

[9] Mainprize T Lipsman N Huang Y Meng Y Bethune A Ironside S Blood-brain barrier opening in primary brain tumors with non-invasive MR-guided focused ultrasound: a clinical safety and feasibility study. Sci Rep. (2019) 9(1):321. 10.1038/s41598-018-36340-0 30674905 PMC6344541

[10] Park SH Kim MJ Jung HH Chang WS Choi HS Rachmilevitch I One-year outcome of multiple blood-brain barrier disruptions with temozolomide for the treatment of glioblastoma. Front Oncol. (2020) 10:1663. 10.3389/fonc.2020.01663 33014832 PMC7511634

[11] Meng Y Reilly RM Pezo RC Trudeau M Sahgal A Singnurkar A MR-guided focused ultrasound enhances delivery of trastuzumab to Her2-positive brain metastases. Sci Transl Med. (2021) 13(615):eabj4011. 10.1126/scitranslmed.abj4011 34644145

[12] Abrahao A Meng Y Llinas M Huang Y Hamani C Mainprize T First-in-human trial of blood-brain barrier opening in amyotrophic lateral sclerosis using MR-guided focused ultrasound. Nat Commun. (2019) 10(1):4373. 10.1038/s41467-019-12426-9 31558719 PMC6763482

[13] Gasca-Salas C Fernández-Rodríguez B Pineda-Pardo JA Rodríguez-Rojas R Obeso I Hernández-Fernández F Blood-brain barrier opening with focused ultrasound in Parkinson’s disease dementia. Nat Commun. (2021) 12(1):779. 10.1038/s41467-021-21022-9 33536430 PMC7859400

[14] Bakker A Ixkes AE Venugopal H Ries MG Lak NSM de Vos F Focused ultrasound-enhanced liquid biopsy: a promising diagnostic tool for brain tumor patients. Cancers (Basel). (2024) 16(8):1576. 10.3390/cancers16081576 38672658 PMC11049441

[15] Yuan J Xu L Chien C-Y Yang Y Yue Y Fadera S First-in-human prospective trial of sonobiopsy in high-grade glioma patients using neuronavigation-guided focused ultrasound. NPJ Precis Oncol. (2023) 7(1):92. 10.1038/s41698-023-00448-y 37717084 PMC10505140

[16] Belykh E Miller EJ Carotenuto A Patel AA Cavallo C Martirosyan NL Progress in confocal laser endomicroscopy for neurosurgery and technical nuances for brain tumor imaging with fluorescein. Front Oncol. (2019) 9:554. 10.3389/fonc.2019.00554 31334106 PMC6616132

[17] Belykh E Zhao X Ngo B Farhadi DS Kindelin A Ahmad S Visualization of brain microvasculature and blood flow *in vivo*: feasibility study using confocal laser endomicroscopy. Microcirculation. (2021) 28(3):e12678. 10.1111/micc.12678 33426724

[18] Hynynen K McDannold N Vykhodtseva N Jolesz FA. Noninvasive MR imaging-guided focal opening of the blood-brain barrier in rabbits. Radiology. (2001) 220(3):640–6. 10.1148/radiol.2202001804 11526261

[19] Gaur P Casey KM Kubanek J Li N Mohammadjavadi M Saenz Y Histologic safety of transcranial focused ultrasound neuromodulation and magnetic resonance acoustic radiation force imaging in rhesus macaques and sheep. Brain Stimul. (2020) 13(3):804–14. 10.1016/j.brs.2020.02.017 32289711 PMC7196031

